# Determining the Impact of Histology on the Incidence, Pattern, and Timing of Recurrences in Patients with Renal Cell Carcinoma: A Pooled Analysis from the SORCE and ASSURE Trials

**DOI:** 10.1016/j.euros.2025.07.003

**Published:** 2025-07-26

**Authors:** Bhavna Oza, Eleni Frangou, Tim Eisen, Grant D. Stewart, Axel Bex, David Harrison, Mahesh K.B. Parmar, Ruth Langley, Duncan Gilbert, Angela Meade

**Affiliations:** a MRC Clinical Trials Unit at UCL, Institute of Clinical Trials & Methodology, London, UK; b Department of Oncology, Cambridge University Hospitals NHS Foundation Trust, Addenbrooke’s Hospital, Cambridge Biomedical Campus, Cambridge, UK; c Department of Surgery, Addenbrooke’s Hospital, Cambridge Biomedical Campus, University of Cambridge, Cambridge, UK; d UCL Division of Surgery and Interventional Science, Royal Free London NHS Foundation Trust, London, UK; e Netherlands Cancer Institute, Amsterdam, The Netherlands; f School of Medicine, University of St Andrews, St Andrews, UK

**Keywords:** Renal cell carcinoma, Clear cell, Papillary, Chromophobe, Sarcomatoid, SORCE, ASSURE, Recurrence patterns

## Abstract

**Background and objective:**

Outcomes after nephrectomy for intermediate- and high-risk renal cell carcinoma (RCC) according to histological subtype are poorly characterised. This study aims to determine the value of RCC histology in predicting survival and to inform on surveillance strategies in relation to patterns of first recurrence.

**Methods:**

We pooled data from phase 3 trials: SORCE (*n* = 1689) and ASSURE (*n* = 1853). Of 3542 patients, 2881 had clear-cell RCC (ccRCC), 269 had papillary RCC (pRCC), 201 had chromophobe RCC (chRCC), and 191 had sarcomatoid RCC (sRCC). Relapse rates, median time to relapse (TTR), and first relapse sites were reported. Multivariable Cox regression models evaluated overall survival by histology, adjusting for initial relapse location and other important clinical factors.

**Key findings and limitations:**

Patients with pRCC and ccRCC had similar overall survival (log-rank *p* = 0.1). The median TTR for those with pRCC was 1.34 yr (interquartile range [IQR] 0.76, 2.59) compared with 1.78 yr (IQR 0.96, 3.38) for ccRCC patients (*p* = 0.012). Patients with chRCC had a median TTR of 2.72 yr (IQR 0.91, 4.11), and those with sRCC had a median TTR of 0.74 yr (IQR 0.50, 1.55). For sRCC patients, relapsing in the chest was associated with a lower risk of death than those relapsing in the abdomen (hazard ratio [HR] 0.5, confidence interval [CI]: 0.3, 0.88; *p* = 0.06). A similar trend was shown for pRCC (HR 0.5, CI: 0.2, 1.3; *p* = 0.1). Recurrence patterns for World Health Organization 2020 molecularly classified RCCs were not included. Despite pooling phase three datasets, small event numbers led to imprecise estimates, particularly for chRCC.

**Conclusions and clinical implications:**

Patients with intermediate and high-risk pRCC relapse earlier than those with ccRCC. Papillary RCC and sRCC first recurring in the abdomen exhibit poor prognosis, warranting consideration of additional abdominal imaging to enhance early relapse detection. ChRCC exhibits favourable prognosis and could avoid image-based surveillance until year 2.

**Patient summary:**

This study evaluates pooled data from large phase 3 trials to precisely delineate relapse patterns for patients with intermediate- and high-risk cell renal cell carcinoma (RCC) according to their histology. The site and timing of first relapse provide useful information to support histology-specific RCC surveillance after nephrectomy. Development of genetic and molecular signatures corresponding to relapses at poor prognosis sites for each histology will individualise follow-up and is the next step.

## Take Home Message

Distinct relapse patterns of patients with higher-risk renal cell carcinoma (RCC) according to histology were shown, using pooled phase 3 trial data. RCC subtype-specific surveillance protocols should be developed. Analyses of RCC tumours to identify histology-specific genetic and molecular markers of relapse comprise the next step.

## 1 Introduction

Non–clear cell renal cell carcinoma (non-ccRCC) accounts for 15–25% [1] of primary renal malignancies, of which papillary (pRCC) and chromophobe (chRCC) renal cell carcinoma (RCC) subtypes comprise the majority (80% of non-ccRCC cases [1,2]). Identification of RCC cases with sarcomatoid features (sRCC), which account for ∼5% of RCC cases, is important clinically because of their associated poor prognosis [2,3].

A recent consensus from the British Association of Urological Surgeons (BAUS) for risk-stratified surveillance after nephrectomy for resectable RCC is based on a synthesis of international guidance [[4], [5], [6]] largely supported by ccRCC data [7]. BAUS promotes a unified low-, intermediate-, and high-risk surveillance strategy for, respectively, ccRCC, pRCC, and chRCC referencing subtype-specific prognostic scores to define risk groups [[8], [9], [10], [11]]. There is no specific guidance for sRCC. High-level evidence supporting a unified surveillance protocol for the major RCC histologies is lacking. In 2021, the RECUR consortium [12] examined recurrence data from a large retrospective European cohort of radically treated patients with RCC. It showed that those with pRCC relapsed sooner (median time to relapse [TTR] 19 mo [interquartile range {IQR} 8.6–41.1]) than those with ccRCC (median TTR 21.2 mo [IQR 7.9–41.1]). Only 1.7% of patients with pRCC relapsed beyond 5 yr compared with 13.5% of patents with ccRCC. Of the patients with chRCC, 18.2% recurred after 5 yr, and the median TTR was 37.4 mo (IQR 11.1–64.6) [12]. To better reflect their variable relapse behaviours, the authors proposed the development of subtype-specific surveillance guidance. They suggested biannual abdominal imaging in the first 2 yr after nephrectomy for patients with pRCC to optimise early detection of relapses and to consider avoiding imaging-based surveillance for patients with chRCC given their favourable prognosis overall.

In 2022, based on the data from Keynote-564 [13], adjuvant pembrolizumab became available for patients with higher-risk RCC, including those with resected oligometastatic non-ccRCC. This sparked renewed focus on optimising postnephrectomy surveillance, in order to detect potentially resectable relapses early. In this study, we characterise the precise relapse patterns of patients with non-ccRCC, focusing on those with intermediate and high risks of recurrence, using data from two large phase 3 trials. It is the first study to include a cohort of patients with sRCC.

SORCE (NCT00492258) [14] and ASSURE (NCT00326898) [15] were two international randomised controlled trials that investigated adjuvant tyrosine kinase inhibitors for patients with initially localised RCC after nephrectomy. Although neither of the trials met its primary endpoint, these provided a rich data source for examining the relapse behaviours of patients with intermediate- and high-risk RCC. We pooled data from the trials and compared patients with pRCC, chRCC, and sRCC with patients with ccRCC. Patients with a low risk were not recruited to either trial because they are usually cured by surgery or ablation and were therefore not assessed in this study.

We evaluated the TTR after nephrectomy and the impact of location of first metastases on survival for each cohort with the primary aim of gaining high-level understanding of their relapse patterns. The second aim was to provide further information to support the development of subtype-specific postnephrectomy surveillance.

## 2 Patients and methods

### 2.1 Patients

The combined dataset consisted of patients who had undergone curative nephrectomy (partial or total) and had been randomised to either SORCE [14] or ASSURE [15]. SORCE (NCT00492258) evaluated up to 3 yr of adjuvant sorafenib compared with placebo and recruited intermediate- and high-risk (2003 Leibovich score >3) participants between 2007 and 2011 from 147 centres in Europe and Australia. ASSURE (NCT00326898) assessed a year of adjuvant sunitinib or sorafenib, and recruited intermediate- and high-risk participants (>pT1b, N_any_) between 2005 and 2011 from 226 centres in North America and Canada. Data from SORCE were available via application to the SORCE executive committee, and ASSURE data were collected from the NCTN-NCORP data archive.

The patients had performance status 0–1 (Supplementary Table 2), were without prior malignancies, and aged >18 yr, with no evidence of residual macroscopic disease on postoperative computed tomography. Histological diagnosis followed protocolled guidance, without a central review.

The pooled dataset was stratified into cohorts of patients with ccRCC, pRCC, chRCC, and sRCC. In keeping with the findings from the SORCE and ASSURE trials, there was no significant difference in either disease-free (DFS) or overall (OS) survival between the treatment and placebo groups in patients within the four cohorts for either trial (Supplementary Fig. 2 and Supplementary Table 1). To maximise the sample size, participant data from the whole cohort were included in this study. Participants with mixed histology (that did not include a sarcomatoid component) and those with other histologies were excluded.

The 2003 Leibovich score components [16] were prospectively collected in both trials. The 2003 Leibovich score has shown good discrimination between intermediate- and high-risk ccRCC and non-ccRCC cohorts; therefore, we used it to risk stratify all patients in this study [17].

### 2.2 Outcome measurements

DFS was defined as the interval from randomisation to first local recurrence, distant metastasis, new primary RCC, or death from any cause. OS was the time from randomisation to death from any cause, including RCC. Median TTR was the time (in years) at which 50% of the patients experienced a relapse; the starting point was the date of surgery and the exit date was the date of the first event.

Baseline patient data common to both trials were reported by histology. For each histology, sites of recurrence by organ site were summarised, using absolute numbers and percentages and graphically. The relapse rate included those having at least one local or distant first recurrence. Local sites were specified as renal bed in ASSURE and as renal bed, remnant kidney, and local node in SORCE. Organ sites of relapse obtained from the trial dataset were sorted manually into three localised categories: “chest”, “abdominal”, and “other”. For the location-based analysis, the categories were “chest only”, “abdomen only”, and “chest and abdomen”.

### 2.3 Statistical analysis

Baseline characteristics are presented as mean (range) or median (IQR) for continuous variables and frequencies with percentages for categorical variables. The log-rank test was used to compare the survival distributions between histological subtypes (clear cell served as the reference group). Patients not experiencing an event were censored at the last time seen on the trial.

Multivariable Cox regression models for OS, adjusting for age, sex, T stage, N stage, performance score, study indicator, type of nephrectomy, and site of relapse indicator, were fitted for each histology. The site of relapse had three groups (“abdomen”, “chest”, and “abdomen and chest”). The abdomen group was defined as the reference group.

The hazard ratios (HRs) and associated 95% confidence intervals (CIs) are presented. All statistical tests were two sided. Analyses were performed in Stata 18 (Stata Corporation, College Station, TX, USA).

## 3 Results

The overall cohort for this analysis comprised 3542 patients in total; 1689 had participated in SORCE and 1853 in ASSURE. Of the total patients, 38% (1329/3542) had an intermediate risk of relapse by 2003 Leibovich criteria and 52% (1850/3542) had a high risk. Of 3542 patients, 2881 (81%) had ccRCC, 269 (8%) pRCC, 201 (6%) chRCC, and 191 (5%) sRCC. Out of the 3542 participants, 2197 did not experience an event of interest. Table 1 shows the clinical/pathological and surgical features of the combined cohort, stratified by histological subtype.

**Table 1:** Baseline characteristics of participants in the pooled sample stratified by RCC histological subtype

Variable at baseline	Clear cell (*N* = 2881, 81%)	Papillary (*N* = 269, 8%)	Chromophobe (*N* = 201, 6%)	Sarcomatoid (*N* = 191, 5%)
Age at randomisation, yr, mean (range)	57 (20–86)	58 (19–80)	52(24–84)	57 (27–83)
Sex, *n* (%)				
Female	890 (31)	59 (22)	84 (42)	58 (30)
Performance status a, *n* (%)				
0	2305 (81)	213 (79)	167 (84)	140 (74)
1	529 (19)	54 (20)	31 (16)	47 (25)
2	2 (<1)		0 (0)	1 (1)
3	2 (<1)	1 (1)	0 (0)	0 (0)
Pathological T stage of primary tumour, *n* (%)				
pT1a	10 (<1)	2 (<1)	1 (<1)	2 (1)
pT1b	285 (10)	28 (11)	10 (5)	10 (5)
pT2	661 (23)	92 (35)	100 (51)	44 (24)
pT3a-4	1898 (67)	143 (54)	86 (44)	131 (70)
Regional lymph node status, *n* (%)				
pN1/ pN2	114 (4)	54 (20)	12 (6)	41 (21)
Tumour size, *n* (%)				
≥10 cm	776 (30)	88 (36)	86 (48)	74 (47)
Nuclear grade b, *n* (%)				
1 or 2	967 (34)	99 (37)	77 (40)	12 (6)
3	1432 (50)	143 (54)	96 (49)	35 (19)
4	480 (16)	23 (9)	21 (11)	142 (75)
Histological tumour necrosis, *n* (%)				
Yes	1209 (45)	158 (63)	72 (39)	117 (73)
Leibovich risk group c, *n* (%)				
High	1468 (56)	147 (61)	87 (50)	148 (95)
Type of nephrectomy, *n* (%)				
Partial	116 (4)	29 (11)	5 (3)	9 (5)
Type of operation, *n* (%)				
Laparoscopic	1234 (44)	109 (41)	85 (43)	73 (38)
Follow-up time for survivors (yr), median (IQR)	6.0 (4.5, 7.2)	6.0 (4.7, 7.1)	6.0 (4.7, 7.1)	5.4 (4.4, 6.4)

ECOG = Eastern Cooperative Oncology Group; IQR = interquartile range; ISUP = International Society of Urological Pathology; RCC = renal cell carcinoma; WHO = World Health Organization.

a The SORCE trial used ECOG performance status. ASSURE used WHO performance status (Supplementary Table 1).

b The SORCE grading system simplifies and selects the worst ISUP features at each grade. ASSURE used Fuhrman grading system. For details of grading components, see Supplementary Table 2.

c The 2003 Leibovich scores were derived from baseline histological data provided in the ASSURE dataset.

### 3.1 DFS and OS

**Fig. 1:**
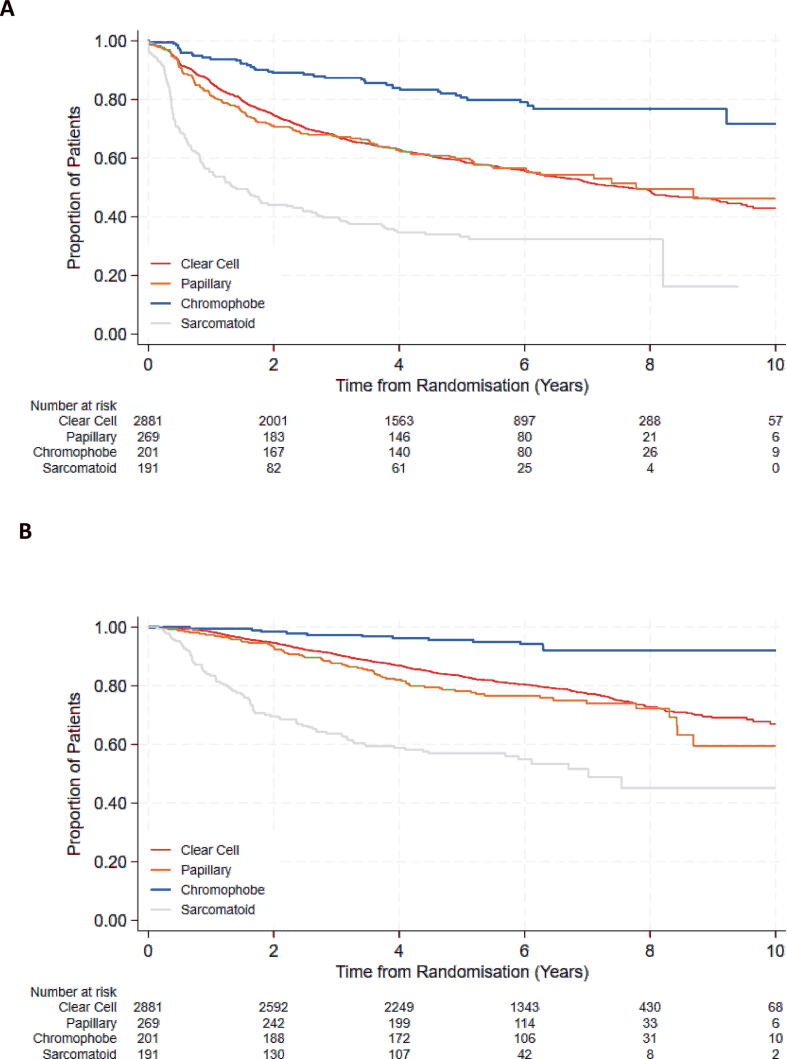
(A) DFS and (B) OS for patients with papillary RCC, chromophobe RCC, and sarcomatoid RCC compared with those for patients with clear-cell RCC. DFS = disease-free survival; OS = overall survival; RCC = renal cell carcinoma.

The median DFS for patients with pRCC was 7.78 yr (IQR 1.56, not reached [NR]), similar to that observed for patients with ccRCC: 7.82 yr (IQR 1.84, NR; log-rank *p* > 0.9; Fig. 1A). Patients with chRCC exhibited favourable DFS (log-rank *p* < 0.001) compared with those with ccRCC, with median DFS not reached (IQR 9.22, NR). Patients with sRCC had the shortest median DFS (log-rank *p* < 0.001) of 1.40 yr (IQR 0.40, 8.20; Fig. 1A).

The median OS for the pRCC cohort was not reached (IQR 6.99, NR) and not statistically different from that of the ccRCC cohort; the median OS was 11.36 yr (IQR 7.54, NR; log-rank *p* = 0.1). OS was poorest for patients with sRCC (median OS 7.02 yr, IQR 1.61, NR; log-rank *p* < 0.001) and highest for patients with chRCC (median OS not reached; log-rank *p* < 0.001; Fig. 1B).

The 2-yr DFS rates were 75% (95% CI: 73–76%) for ccRCC, 71% (95% CI: 65%, 76%) for pRCC, 89% (95% CI: 84%, 93%) for chRCC, and 44% (95% CI: 37%, 51%) for sRCC. By year 5, the DFS rates were 59% (95% CI: 57%, 61%) for ccRCC, 60% (95% CI: 54%, 66%) for pRCC, 81% (95% CI: 74%, 86%) for chRCC, and 33% (95% CI: 26%, 40%) for sRCC.

### 3.2 Relapse summaries according to subtype

For patients with pRCC, the median TTR after nephrectomy was 1.34 yr (IQR 0.76, 2.59), which was 5.3 mo less than the median TTR for those with ccRCC (median TTR 1.78 yr, IQR 0.96, 3.38; *p* = 0.012). The median TTR was longest for chRCC at 2.72 yr (IQR 0.91, 4.11) and shortest for sRCC at 0.74 yr (IQR 0.5, 1.55) after nephrectomy.

**Fig. 2:**
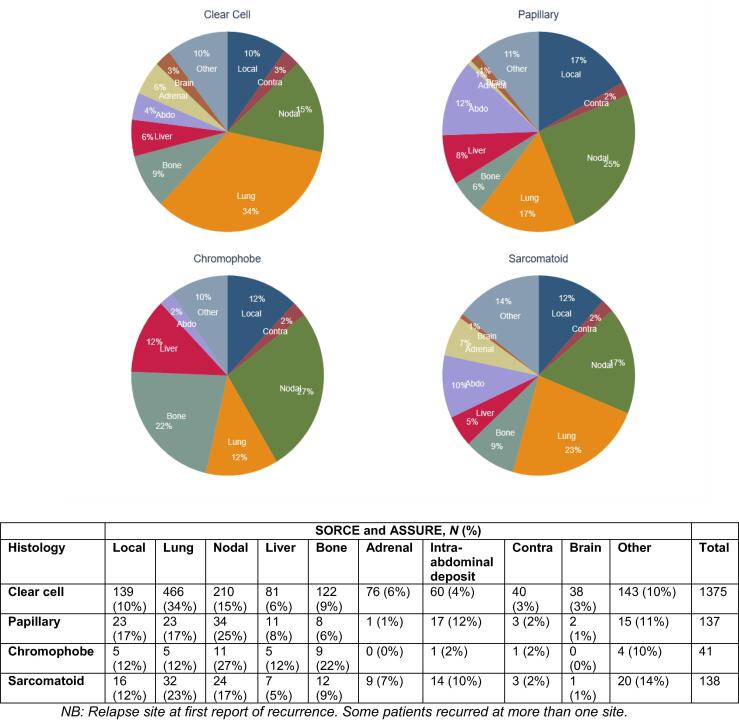
Relapse site at first recurrence by histological subtype. Abdo = abdomen; Contra = contralateral.

The lung, nodes, bone, and liver were the commonest single sites of relapse amongst the four histological subtypes (Fig. 2). Distant lymph node relapses (including those in the abdomen and chest) were the commonest sites of relapse for all non–clear cell subtypes: pRCC (25%, 34/137), chRCC (27%, 11/41), and sRCC (17%, 24/138; Fig. 2). Of note, distant nodal relapses in patients with pRCC were associated with worse 5-yr OS (36%, 95% CI: 20%, 52%) than in those with ccRCC (61%, 95% CI: 54%, 67%). Sarcomatoid RCC patients with distant lymph-node relapses had the worst 5-yr OS of 19% (95% CI: 6%, 37%). Relapses to the lungs, for those with chRCC, although small in number, were associated with poorest 5-yr OS: 33% for chRCC (95% CI: 1%, 75%) compared with 71% for ccRCC (95% C:; 66%, 75%), 61% for pRCC (95% CI: 20%, 52%), and 51% for sRCC (95% CI: 6%, 37%).

### 3.3 OS according to the location of relapse

For patients with pRCC, relapses first in the abdomen were associated with a worse 5-yr OS rate of 46% (95% CI: 31%, 59%) compared with 61% (95% CI: 32%, 80%) for those relapsing first in the chest. A similar pattern was shown for those with sRCC in whom the 5-yr OS rate for relapses first in the chest was 51% (95% CI: 35%, 67%) compared with 26% (95% CI: 15%, 37%) for relapses in the abdomen. The converse was true for those with chRCC; the 5-yr OS rate for chest relapses was 33% (95% CI: 1%, 77%) compared with 78% (95% CI: 47–93%) for abdominal relapses, although the numbers were small for this analysis.

For patients with sRCC, relapsing first in the chest was associated with a lower risk of death than relapsing first in the abdomen (HR 0.5, CI: 0.3, 0.88; *p* = 0.06). A similar trend was shown for patients with pRCC (HR 0.5, CI: 0.2, 1.3; *p* = 0.1).

The number of patients with chRCC and those progressing simultaneously in the abdomen and chest were too small to draw meaningful conclusions from the models. The multivariable Cox regression models for OS are detailed in Supplementary Table 3.

## 4 Discussion

This study represents the largest contemporary analysis of prospectively collected phase 3 trial data from patients with intermediate- and high-risk pRCC, chRCC, and sRCC directly compared with those from patients with ccRCC. We show that survival of patients with pRCC and ccRCC are similar (log-rank *p* = 0.110). Of the cohorts examined, patients with chRCC have the best and patients with sRCC have the poorest outcomes, which is broadly consistent with the literature [12,[18], [19], [20], [21]].

Patients with pRCC exhibited worse median TTR, DFS, and OS than those previously reported for the subtype [12,22]. In previous studies, patients with pRCC had better survival than patients with chRCC [23,24]. One large cohort of 2237 patients showed papillary subtype to be an independent predictor of improved 5-yr relapse-free survival (HR 0.30, 95% CI: 0.09–0.97), though all-cause mortality was higher (HR 1.60, 95% CI: 0.89–2.81) [24]. The majority of patients in the cohort had undergone partial (75%) nephrectomy for mostly T1 (70%) cancers, whereas our study focused on higher-risk patients (all Leibovich score >3 and T3a-4 64%) who underwent total nephrectomy (94%). Our pRCC cohort exhibited histological features similar to those with sRCC (Table 1), including high rates of baseline nodal involvement and coagulative tumour necrosis, which may link directly to their poorer outcomes.

Our study highlights the prognostic utility of first relapse sites for patients with RCC. Patients with pRCC and sRCC who relapsed first in the abdomen were associated with worse survival than those relapsing in the chest. Relapses in the nonregional lymph nodes conferred particularly poor survival in our pRCC and sRCC groups. Furthermore, relapses to nonregional lymph nodes were proportionally more numerous than relapses to other sites in all the non-ccRCC histological subtypes (Fig. 2). In the pRCC cohort, metastases found in distant nodes were proportionally higher in patients with baseline nodal involvement: 28% (15/54) of patients with N1+ disease versus 10% (22/215) of patients with N0 disease. The tendency towards lymphatic spread in patients with pRCC and its associated poor prognosis have been noted previously [12,25,26], highlighting the need to precisely delineate the anatomy of nodal spread in patients with pRCC. A sentinel lymph node analysis or locoregional lymph node dissection at the time of nephrectomy for selected patients with high-risk pRCC has been proposed [25,27,28].

Although conclusions about our chRCC cohort are limited by small numbers, those with chRCC relapsing first in the chest were associated with an unfavourable prognosis compared with those relapsing first in the abdomen (33% vs 78% 5-yr OS rate), and there was a trend towards the worst survival for chest relapses in chRCC cases compared with chest relapses in the other three subtypes. This unique pattern of behaviour for chRCC was noted previously by Dudani et al [18] and should be explored further as it may reflect true differences in the biology of chRCC.

Detection of metastases while still potentially resectable has become of critical importance, given the recent availability of adjuvant pembrolizumab after metastasectomy in patients with ccRCC and non-ccRCC. As such, with distinct relapse behaviours shown in patients with intermediate- and high-risk pRCC, chRCC, and sRCC, our data support a clear role for histology-specific surveillance strategies. For example, for patients with pRCC and sRCC in whom the median TTR was <2 yr after nephrectomy, we support the need for enhanced surveillance, particularly in the abdomen, in the first 2 yr after nephrectomy [12].

Patients with chRCC had the best prognosis overall and a median TTR of 2.72 yr. It may therefore be possible to avoid imaging in patients with chRCC in the first 2 yr unless clinically indicated. Given the high rates of bone metastases in patients with chRCC, selected use of bone scans in their surveillance may be of value.

Current understanding of the molecular correlates of prognosis in patients with RCC is evolving [29,30] and requires development to support their use in individualising follow-up for patients with non-ccRCC. Longitudinal sampling of non–clear cell tumours collected in TRANSORCE (in which nephrectomy samples were collected from 1500 SORCE trial participants) will be informative. Findings from our study have directly informed on cohorts of interest, notably patients with pRCC and sRCC relapsing first in the abdomen and those relapsing in distant nodes. Rapid whole slide scanning technology and high-quality three-dimensional digital imaging alongside immune-histological and gene transcription panelling, to explore tumour tissue for potential molecular markers for further testing, are underway.

There are several limitations of this study. Firstly, patients with poor performance and those relapsing within 90 d of nephrectomy were not represented due to the design of the trials. Secondly, neither SORCE nor ASSURE collected data to define RCC categories molecularly in line with World Health Organization 2022 classification. This will be important going forward, as we learn more about how to individualise the treatment options. Despite pooling data from large datasets, in some cases, small event numbers led to imprecise statistical estimates, particularly for the chRCC group. There were very few events in the stratified Kaplan-Meier survival analysis according to recurrence sites to carry out further statistical analysis for any of the histologies. Finally, pooling data from trials with different protocols led to variability in the capture of some data between the trials, for example, reporting of sarcomatoid histology and relapse sites between the trials. Furthermore, follow-up time intervals and modes of radiology were different in the SORCE and ASSURE protocols, which limits comparability of relapse detection between trials. However, overall, the use of protocol-driven follow-up data vastly reduced missing data and also minimised data point variability within the datasets. Future prospectively designed collaborative analyses that unify protocol schedules between trials should be considered.

## 5 Conclusions

In RCC, histological subtype remains an important predictor of relapse behaviour and survival. Patients with intermediate/high-risk pRCC exhibit poorer prognosis than expected, particularly those relapsing in abdominal and nodal sites. Subtype-specific surveillance guidance based on the unique relapse patterns of patients with non-ccRCC may improve on relapse detection and could avoid excessive imaging frequency and duration. Development of genetic and molecular signatures corresponding to relapses at poor prognosis sites for each histology will further individualise follow-up and is the next step.

  ***Author contributions*:** Bhavna Oza and Elena Frangou have full access to all the data in the study and takes responsibility for the integrity of the data and the accuracy of the data analysis.

  *Study concept and design*: Oza, Frangou, Meade, Gilbert.

*Acquisition of data*: Frangou.

*Analysis and interpretation of data*: Oza, Frangou.

*Drafting of the manuscript*: Oza, Frangou.

*Critical revision of the manuscript for important intellectual content*: All authors.

*Statistical analysis*: Frangou.

*Obtaining funding*: None.

*Administrative, technical, or material support*: None.

*Supervision*: Meade, Langley, Gilbert.

*Other*: None.

  ***Financial disclosures:*** Bhavna Oza certifies that all conflicts of interest, including specific financial interests and relationships and affiliations relevant to the subject matter or materials discussed in the manuscript (eg, employment/affiliation, grants or funding, consultancies, honoraria, stock ownership or options, expert testimony, royalties, or patents filed, received, or pending), are the following: Grant D. Stewart has received educational grants from Pfizer, AstraZeneca, and Intuitive Surgical; has received consultancy fees from Pfizer, MSD, EUSA Pharma, and CMR Surgical; has received travel expenses from MSD and Pfizer; has received speaker fees from Pfizer; and reports being a clinical lead (urology) National Kidney Cancer Audit and topic advisor for the NICE kidney cancer guideline. Tim Eisen reports a leadership or fiduciary role in other board, society, committee, or advocacy group, paid or unpaid (Macmillan Cancer Support, trustee for 10 yr till 2021; Cambridge University Health Partners, nonexecutive director; and DeviceChooser Ltd Director); stock or stock options with AstraZeneca (self and spouse) and Roche (self); receipt of equipment, materials, drugs, medical writing, gifts, or other services from AstraZeneca (research support); and other: AstraZeneca (till March 2020)—employment as VP Oncology Early Clinical Development, and Roche (from March 2020)—employment as VP GU and GI Oncology Late Clinical Development. The remaining authors have nothing to disclose.

  ***Funding/Support and role of the sponsor*:** None.

  ***Acknowledgments*:** We are grateful to the members of the SORCE trial management group: Tim Eisen, Eleni Frangou, Bhavna Oza, Grant D. Stewart, Axel Bex, Alastair Ritchie, Rick Kaplan, Benjamin Smith, Ian Davis, Martin R. Stockler, Laurence Albiges, Bernard Escudier, James Larkin, Steven Joniau, Barry Hancock, Gregers Hermann, Joaqium Bellmunt, Mahesh K.B. Parmar, Patrick Royston, and Angela Meade. We thank Professor Naomi B. Haas and our colleagues in the ASSURE study trial management group for providing us with support and invaluable clinical data: Judith Manola, Robert G. Uzzo, Keith T. Flaherty, Christopher G. Wood, Christopher Kane, Michael Jewett, Janice P. Dutcher, Michael B. Atkins, Michael Pins, George Wilding, David Cella, Lynne Wagner, Surena Matin, Timothy M. Kuzel, Wade J. Sexton, Yu-Ning Wong, Toni K. Choueiri, Roberto Pili, Igor Puzanov, Manish Kohli, Walter Stadler, Michael Carducci, Robert Coomes, and Robert S. DiPaola. We are especially grateful to the participants who took part in the SORCE and ASSURE trials. This manuscript was prepared using data from Datasets NCT00326898-1 to NCT00326898–5 and NCT00326898-7 from the NCTN Data Archive of the National Cancer Institute’s (NCI’s) National Clinical Trials Network (NCTN). Data were originally collected from clinical trial number NCT00326898 (ASSURE). All analyses and conclusions in this manuscript are the sole responsibility of the authors and do not necessarily reflect the opinions or views of the clinical trial investigators, the NCTN, or the NCI.
