# Supplementary material for: Determining the Impact of Histology on the Incidence, Pattern, and Timing of Recurrences in Patients with Renal Cell Carcinoma: A Pooled Analysis from the SORCE and ASSURE Trials
Source: Eur Urol Open Sci. 2025 Jul 26;79:19–26. doi: 10.1016/j.euros.2025.07.003 (PMC12314385; doi:10.1016/j.euros.2025.07.003)
Supplement: Supplementary Data 1 [file mmc1.docx]

**Supplementary material**

Supplementary Figure 1 Kaplan-Meier curves for time to recurrence (TTR) by subtype. Horizontal axis is time from nephrectomy in years.


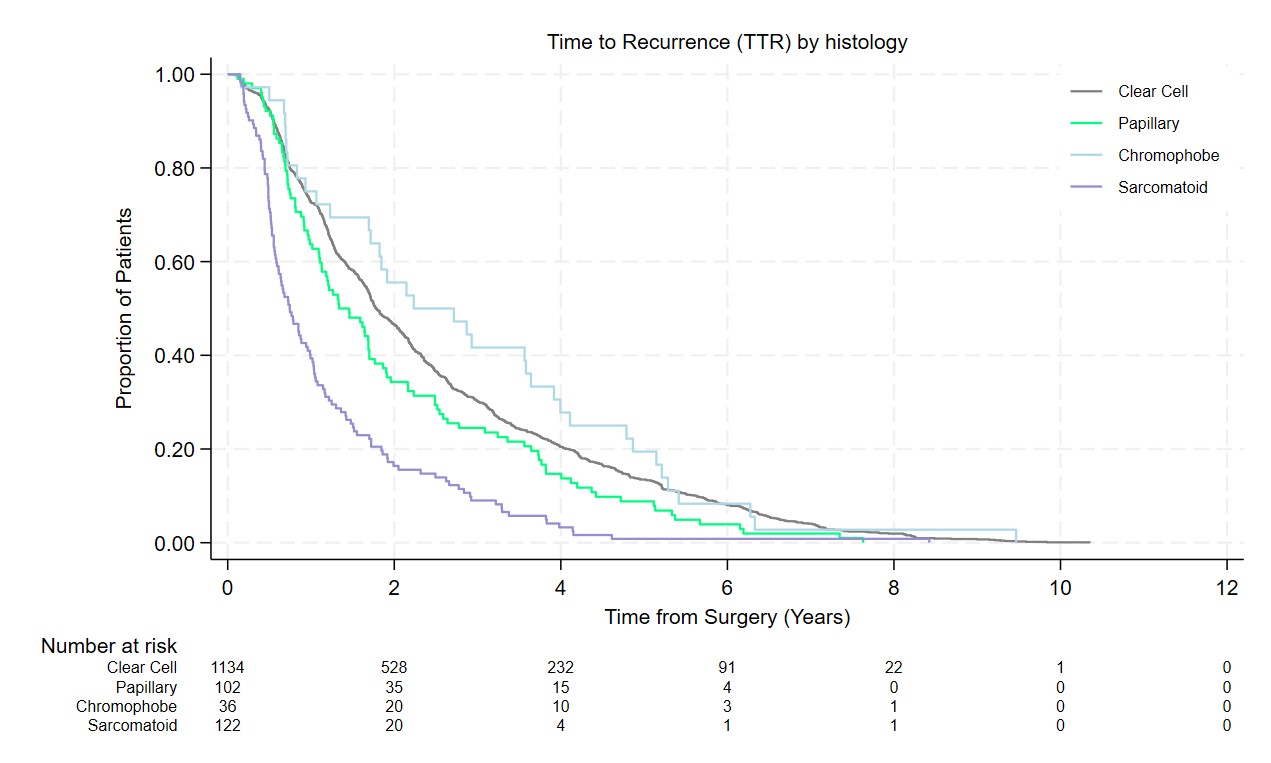


**Supplementary Figure 2: Kaplan Meier curves of pooled SORCE and ASSURE cohorts comparing (i) DFS and (ii) OS in patients receiving TKI treatment and placebo**

**(i)**
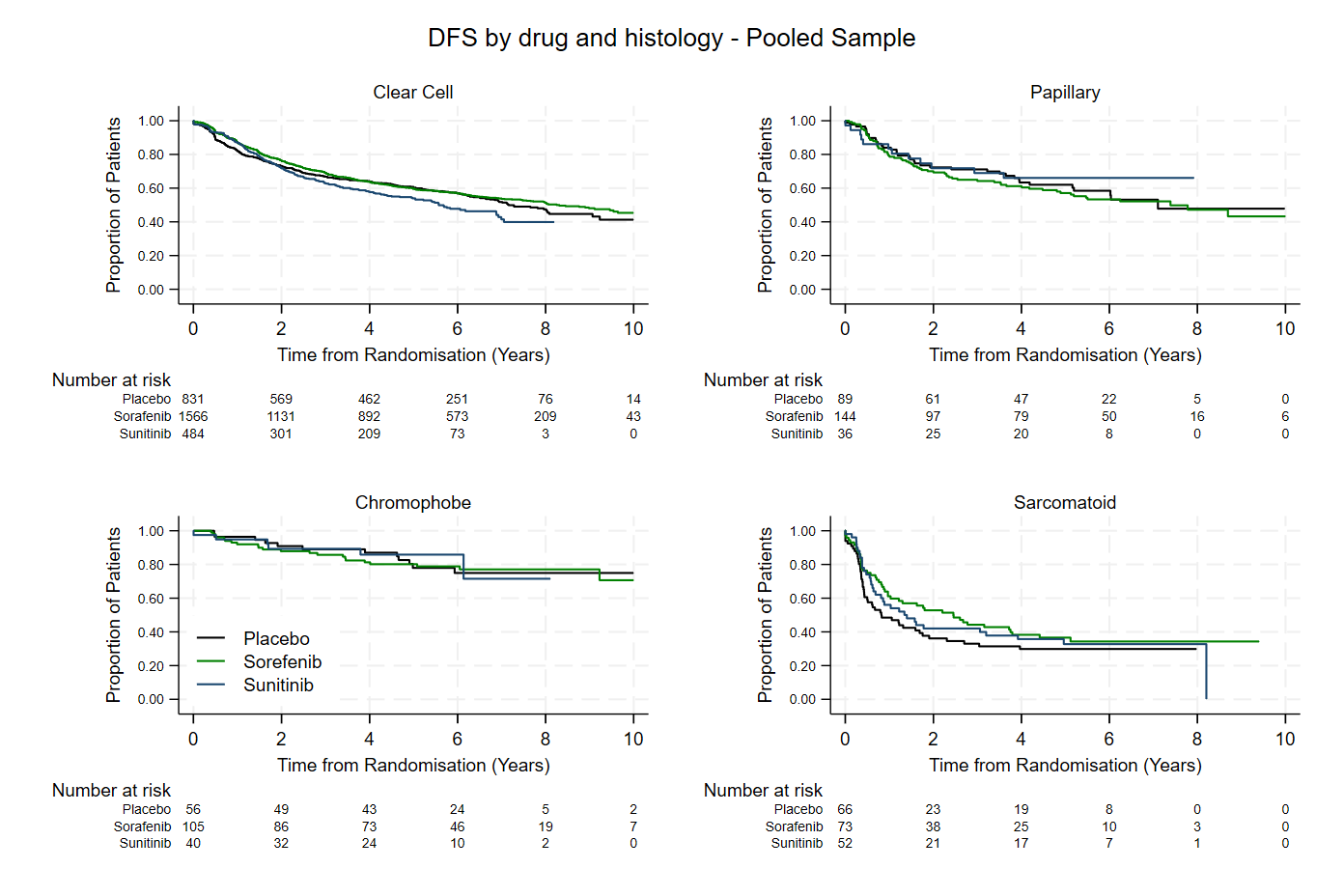


**(ii)**


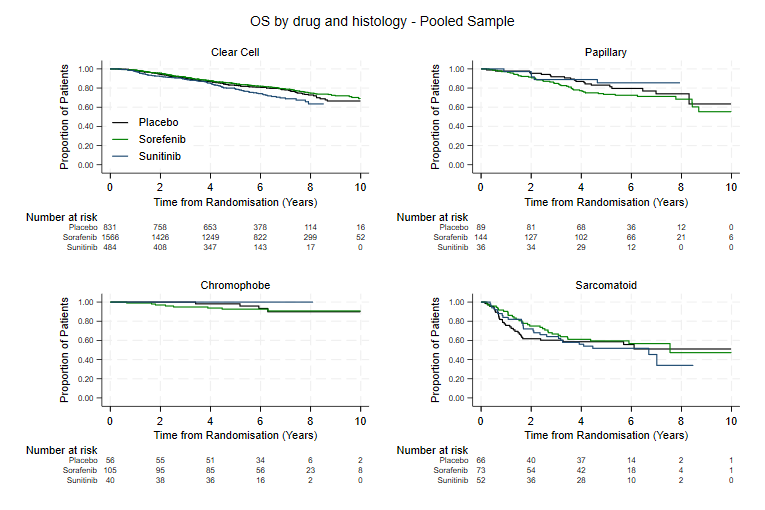


**Supplementary Table 1: post-hoc analysis comparing i. DFS and ii. OS in patients with ccRCC, pRCC, chRCC and sRCC who received TKI treatments-sunitinib or sorafenib versus placebo.**

**Separate analyses were conducted in SORCE and ASSURE datasets, using the log-rank test.**

**DFS**

**SORCE (sorafenib vs placebo)**

| **CC** | **0.8** |
| --- | --- |
| **Pap.** | **0.6** |
| **Chromo.** | **0.97** |
| **Sarco** | **0.5** |

**ASSURE (sorafenib, sunitinib, placebo)**

| **CC** | **0.7** |
| --- | --- |
| **Pap.** | **0.4** |
| **Chromo.** | **>0.9** |
| **Sarco** | **0.6** |

**OS**

**SORCE**

| **CC** | **0.7** |
| --- | --- |
| **Pap.** | **>0.9** |
| **Chromo.** | **0.8** |
| **Sarco** | **0.4** |

**ASSURE**

| **CC** | **0.1** |
| --- | --- |
| **Pap.** | **0.1** |
| **Chromo.** | **0.3** |
| **Sarco** | **0.9** |

### **Supplementary Table 2: Comparison of scales of performance status used to assess patients in SORCE and ASSURE**

| **WHO (SORCE)** | **ECOG (ASSURE)** |
| --- | --- |
| 0: able to carry out all normal activity without restriction | 0: Fully active; no performance restrictions. |
| 1: restricted in strenuous activity but ambulatory and able to carry out light work | 1: Strenuous physical activity restricted; fully ambulatory and able to carry out light work. |
| 2: ambulatory and capable of all self-care but unable to carry out any work activities; up and about more than 50% of waking hours | 2: Capable of all self-care but unable to carry out any work activities. Up and about >50% of waking hours. |
| 3: symptomatic and in a chair or in bed for greater than 50% of the day but not bedridden | Capable of only limited self-care; confined to bed or chair >50% of waking hours. |
| 4: completely disabled; cannot carry out any self-care; totally confined to bed or chair. | Completely disabled; cannot carry out any self-care; totally confined to bed or chair. |

**Supplementary Table 3:** Cox Proportional Hazards Model for each histology exploring overall survival by relapse location. ‘Abdomen’ was the reference group

| **Clear Cell** | **HR** | **95% C.I.** | **p-value** |
| --- | --- | --- | --- |
| **Age** | 1.02 | 1.01, 1.03 | <0.001 |
| **Sex: Female** | 1.3 | 1.0, 1.6 | 0.05 |
| **T stage** | 1.3 | 1.02, 1.5 | 0.03 |
| **N stage: pN1/pN2** | 1.6 | 1.03, 2.4 | 0.04 |
| **Performance status: 1** | 1.5 | 1.1, 1.9 | <0.001 |
| **Study indicator: ASSURE** | 1.3 | 1.05, 1.7 | 0.02 |
| **Nephrectomy: Partial** | 1.01 | 0.5, 2.3 | 0.98 |
| ***Site of relapse*** |  |  |  |
| **Chest** | 0.9 | 0.7, 1.1 | 0.2 |
| **Abdomen and Chest** | 1.6 | 1.0, 2.5 | 0.06 |

| **Papillary** | **HR** | **95% C.I.** | **p-value** |
| --- | --- | --- | --- |
| **Age** | 1.01 | 0.98,1.05 | 0.6 |
| **Sex: Female** | 1.2 | 0.5,2.8 | 0.7 |
| **T stage** | 0.8 | 0.5,1.5 | 0.6 |
| **N stage: pN1/pN2** | 0.6 | 0.3,1.5 | 0.3 |
| **Performance status: 1** | 0.8 | 0.3,1.95 | 0.7 |
| **Study indicator: ASSURE** | 1.1 | 0.5,2.3 | 0.9 |
| ***Site of relapse*** |  |  |  |
| **Chest** | 0.5 | 0.2,1.3 | 0.1 |
| **Abdomen and Chest** | 1.1 | 0.4,3.3 | 0.9 |

*Surgery type was not converging, hence removed from model*

| **Chromophobe** | **HR** | **95% C.I.** | **p-value** |
| --- | --- | --- | --- |
| **Age** | 1.01 | 0.9,1.1 | 0.8 |
| **Sex: Female** | 20.2 | 1.0,398.8 | 0.05 |
| **T stage** | 12.3 | 0.2,804.1 | 0.2 |
| **N stage: pN1/pN2** | 2.5 | 0.3,23.6 | 0.4 |
| **Study indicator: ASSURE** | 0.09 | 0,2.2 | 0.1 |
| **Site of Relapse: Chest** | 0.4 | 0.01,15.9 | 0.6 |

*There were no abdomen/chest relapses; performance score and surgery type were not converging, hence not included in model.*

| **Sarcomatoid** | **HR** | **95% C.I.** | **p-value** |
| --- | --- | --- | --- |
| **Age** | 1.01 | 1.0,1.03 | 0.6 |
| **Sex: Female** | 1.2 | 0.6,2.1 | 0.6 |
| **T stage** | 1.8 | 1.1,2.8 | 0.02 |
| **N stage: pN1/pN2** | 1.9 | 1.1,3.4 | 0.02 |
| **Performance status: 1** | 1.7 | 0.9,3.3 | 0.1 |
| **Study indicator: ASSURE** | 2.1 | 0.6,7.0 | 0.2 |
| **Nephrectomy: Partial** | 0.3 | 0.04,2.3 | 0.2 |
| ***Site of relapse*** |  |  |  |
| **Chest** | 0.5 | 0.3,0.88 | 0.06 |
| **Abdomen and Chest** | 12.3 | 1.1, 133.3 | 0.04 |

*There was only one abdomen/chest relapse hence the ultra-wide confidence interval.*
